# Supplementary material for: Dynamic transcriptomic profiles of zebrafish gills in response to zinc supplementation
Source: BMC Genomics. 2010 Oct 11;11:553. doi: 10.1186/1471-2164-11-553 (PMC3091702; doi:10.1186/1471-2164-11-553)
Supplement: Additional file 2 — Interactive Direct Interaction Network representing the molecular interactions between zinc, copper, iron, calcium and proteins encoded by transcripts changed by zinc supplementation. Mini web-site containing index.html and hyperlinked pages in subdirectory describing a Direct Interaction Network automatically generated based on curated interactions contained within the proprietary PathwayArchitect database. Ovals represent proteins and the circles symbolize metal ions. Objects are coloured by their abundance in zebrafish at the time-point they were significantly different from the control is a scale from -4 fold (dark green) to +4 fold (dark red). Where significant differences were found at more than one time-point, the colour overlay shows expression at the first instance. Dark blue squares denote 'binding', and light blue squares 'expression'; green squares stand for 'regulation', green diamonds for 'metabolism', and green circles for 'promoter binding'. Arrow heads indicate directionality of the interaction where annotated. All nodes and edges can be further interrogated by selecting the relative area of the image. [file 1471-2164-11-553-S2.zip › PathwayArchitect Zn xs DIN/961652.html]

# REGULATION:

|  |  |
| --- | --- |
| Type | REGULATION |
| Effect | Positive |


---

|  |  |
| --- | --- |
| Score | 0 |


---

|  |  |
| --- | --- |
| Reference Count | 7 |


---

|  |  |
| --- | --- |
| Mechanism | Unknown |


---

|  |  |
| --- | --- |
| Reference:0 || Sentence | "These results suggest that DMT1, Fpn1, and Heph are involved in the iron uptake process modulated by copper status." |
| PMID | 11842003 |
| Year | 2002 |
| Species | Human |
| Journal | Am J Physiol Gastrointest Liver Physiol |
| RefScore | 0 |
| Source | PArchNLP |
  |
|  |  |


---

|  |  |
| --- | --- |
 Reference:1 || Sentence | "Iron egress from macrophages is probably also mediated by ferroportin and patients with heterozygous ferroportin mutations develop progressive iron overload in liver macrophages." |
| PMID | 14770366 |
| Year | 2004 |
| Species | Human |
| Journal | Med Sci (Paris) |
| RefScore | 2 |
| Source | PArchNLP |
  ||  |  |


---

|  |  |
| --- | --- |
 Reference:2 || Sentence | "Overexpression of MTP1 in tissue culture cells results in intracellular iron depletion." |
| PMID | 10747949 |
| Year | 2000 |
| Species | Mouse |
|  | Human |
| Journal | J Biol Chem |
| RefScore | 0 |
| Source | PArchNLP |
  ||  |  |


---

|  |  |
| --- | --- |
 Reference:3 || Sentence | "Iron efflux is thought to be mediated by the metal transporter protein, IREG1/ferroportin1/MTP1, and oxidation of Fe(II) to Fe(III) prior to incorporation into fetal transferrin is carried out by the placental copper oxidase." |
| PMID | 11389698 |
| Year | 2001 |
| Species | Rat |
| Journal | Biochem J |
| RefScore | 1 |
| Source | PArchNLP |
  ||  |  |


---

|  |  |
| --- | --- |
 Reference:4 || Sentence | "IREG1 stimulates iron efflux following expression in Xenopus oocytes." |
| PMID | 10882071 |
| Year | 2000 |
| Species | Mouse |
| Journal | Mol Cell |
| RefScore | 0 |
| Source | PArchNLP |
  ||  |  |


---

|  |  |
| --- | --- |
 Reference:5 || Sentence | "Pcm mutants, which are iron deficient at birth, exhibited increased Fpn1-mediated iron uptake and reticuloendothelial iron overload as young adult mice." |
| PMID | 15084469 |
| Year | 2004 |
| Species | Mouse |
| Journal | Development |
| RefScore | 1 |
| Source | PArchNLP |
  ||  |  |


---

|  |  |
| --- | --- |
 Reference:6 || Sentence | RECENT FINDINGS: Hepcidin antimicrobial peptide likely modulates iron transport from macrophages and enterocytes to red blood cell precursors as a consequence of its interaction with SLC40A1/ferroportin, the only known transporter that facilitates iron egress. |
| PMID | 15725899 |
| Year | 2005 |
| Species | Human |
| Journal | Curr Opin Hematol |
| RefScore | 0 |
| Source | PArchNLP |
  |


---

|  |  |
| --- | --- |
